# The impact of cataract progression on accuracy of intraocular lens power measurement

**DOI:** 10.1371/journal.pone.0246816

**Published:** 2021-02-10

**Authors:** Lin Leng, Honglei Li, Min Yin, Han Gao, Ting Shao, Keli Long

**Affiliations:** 1 Qingdao Eye Hospital of Shandong First Medical University, State Key Laboratory Cultivation Base, Shandong Provincial Key Laboratory of Ophthalmology, Shandong Eye Institute, Shandong First Medical University & Shandong Academy of Medical Sciences, Qingdao, Shandong Province, China; 2 Department of Ophthalmology, Affiliated Hospital of Qingdao University, Qingdao, Shandong, China; University of Toronto, CANADA

## Abstract

**Purpose:**

The aim of this study was to assess the impact of cataract progression using the Haigis formula-calculated intraocular lens (IOL) power and investigate the accuracy of IOL power measured at different time points.

**Methods:**

This prospective study was performed on 75 eyes of 75 patients who underwent uneventful cataract surgery. Preoperative ocular parameters including axial length (AL), keratometry (K), anterior chamber depth (ACD), corneal astigmatism, corrected distance visual acuity (CDVA) and uncorrected distance visual acuity (UDVA) examined at the two time points, more than 3 months preoperatively and preoperative 1 day were compared. The ocular parameters measured in the two time points were used to calculate the predicted implanted IOL power and the actual IOL power was chosen on the basis of parameters measured earlier before surgery using the Haigis formula. The mean numerical error (MNE) and mean absolute error (MAE) predicted by the two time points were also compared.

**Results:**

There were significant differences in the ACD, IOL power, UDVA and CDVA (P<0.01), but no statistical differences in AL, mean K and corneal astigmatism (P>0.05) during the average of 5.6 months before surgery. No statistically significant difference was detected in MNE (P>0.05), while the MAE had a significant difference in the two time points (P<0.05).

**Conclusion:**

The IOL power measured earlier before surgery might result in a higher accuracy and the postoperative refractive outcome tended towards emmetropia.

## Introduction

Phacoemulsification is considered to be an effective surgery to cure cataracts, which has evolved to become a refractive procedure [1]. Although the ocular biometry measurement and calculation formulas have been improved, refractive errors still exist with a mean absolute error (MAE) of 0.6 diopters (D) [2–4]. Intraocular lens (IOL) power calculation is a challenge when seeking to achieve favorable postoperative refractive outcomes [5]. It was estimated that 35% of the IOL power calculation error was caused by an effective lens position (ELP) prediction error [4]. Among the existing calculation formulas, the third-generation formulas such as SRK-T, Holladay and Hoffer-Q only measure the axial length (AL) and mean keratometry (K), whereas recent formulas estimate ELP combined with the preoperative ACD measurement, which are more accurate in predicting the postoperative ELP and are more effective in cataract eyes with normal and long AL [6].

Optical biometry has been the gold standard for ocular measurements since the introduction of the IOL-Master (Carl Zeiss Meditec AG, Jena, Germany) [7], however, the ocular parameters can be influenced by the degree of lens turbidity [8–11]. Tetsuo found that the postoperative refractive outcome was affected by cataract density and suggested that an increase in cataract density could cause errors in AL measurement, resulting in poorer postoperative refractive outcomes [8]. Freeman reported that posterior subcapsular cataracts and mature cataracts commonly cause measurement failure, at a rate of 15.9% [12]. Generally, measurement on ocular biometry was not repeated within 3 or 6 months, however, difference in IOL power was observed at different times before surgery. Some patients do not undergo surgery until they present with severe cataract that affects their daily lives, making it difficult to determine accurate interval of preoperative measurements. The purpose of this study was to investigate the impact of cataract progression on accuracy of IOL power measurement.

## Materials and methods

The present study was a prospective comparative study including patients with age-related cataracts who were candidates of phacoemulsification with IOL implantation from April 2018 to July 2019. Patients with different IOL measurements before surgery were recruited and the IOL implanted in the eye was measured more than 3 months pre-operatively. The study was approved by the Ethics Committee and the Institutional Review Boards of the Affiliated Hospital of Qingdao University (no.ChiCTR1800015251). In addition, the study was performed in accordance with the tenets of the Declaration of Helsinki and written informed consent was obtained from all subjects.

Patients with cortical cataract and undergoing phacoemulsification were eligible for inclusion in this study. IOL power was calculated using the Haigis formula, which is a fourth-generation IOL power calculation formula. The study aimed at exploring the impact of cataract progression on the accuracy of the formulation and eliminating the interference of other coexistent pre or postoperative ocular pathology to postoperative vision, therefore, the inclusion and exclusion criteria were strict. Exclusion criteria of patients in our study were as follows: (a) known pathology that could affect visual acuity (history of prior ocular trauma, refractive surgery, diabetic retinopathy, macular degeneration and glaucoma, etc,); (b) irregular corneal astigmatism or corneal astigmatism more than 1.00 D that could impact on lens toricity at different time points; (c) abnormal axial length that could reduce the accuracy of the formula (axial length less than 22.0mm or more than 26.0mm); (d) severe degree of cortical opacity that might affect the ocular biometry parameters (preoperative corrected distance visual acuity (CDVA) worse than 0.5 (log MAR), LOCS III grade P>3.5, and mature cataracts); and (e) nuclear cataracts, posterior subcapsular cataracts, or other types of cataract that could confound study results. All subjects’ postoperative CDVA should also be better than 0.1 (log MAR). However, when both eyes of a patient met the inclusion criteria, only the eye that underwent surgery earlier was included in this study and the second eye was excluded to avoid having 2 genetically identical eyes in the data pool.

Data collection included preoperative and postoperative examinations, and refractive data. Additionally, age, sex and laterality were recorded. Ocular biometry measurements were performed by an experienced examiner (HG) using IOL Master 500 (Carl Zeiss Meditec AG, Jena, Germany), who was unaware of the first result of the IOL measurement. All measurements were taken three times and data was averaged after three reproducible readings were obtained. IOL power was calculated using the Haigis formula with optimized IOL constants from the User Group for Laser Interference Biometry (ULIB).

All patients underwent phacoemulsification cataract surgery by one experienced surgeon (KLL) with a standard stop & chop technique with a sutureless 3.0 mm incision under topical anesthesia. All incisions were made at the 135-degree axis of the cornea. At the end of surgery, an aspherical monofocal IOL (Akreos Mi60; Bausch & Lomb) was implanted in the capsular bag in all cases. Three months after surgery, monocular logMAR acuities for uncorrected distance visual acuity (UDVA) and CDVA were measured for distance (5m). Furthermore, an optometrist (MY) performed the procedure for combining objective refraction with subjective refraction. The refractive outcome was converted to the spherical equivalent (SE) to assess the postoperative error. The refractive prediction error was defined as the measured postoperative SE refraction minus the predicted SE that was calculated using the Haigis formula. The mean numerical error (MNE) and the mean absolute error (MAE) were defined as the arithmetic mean of the prediction errors and the mean of the magnitude of the prediction errors, respectively.

Statistical analysis was performed with SPSS software (version 22.0, IBM Corp.). Sample size was calculated with power and sample size online kit. Ocular parameters of our study between the two time points were compared by a paired t test (for parametric data). All patients were divided into several groups according to the change value of preoperative ACD. Postoperative SE among groups were then compared by one-way analysis of variance. A p value less than 0.05 was considered statistically significant.

## Results

A total of 83 cataract patients (83 eyes) undergoing uneventful phacoemulsification were included in this study. No surgical complications occurred. There were 8 cases (8 eyes) lost to follow-up. Therefore, 75 eyes of 75 patients were analyzed during the recruitment period. Of the 75 patients, 35 (46.7%) were female. The ages of patients ranged from 51 to 75 years (66.16±10.37 years) and the first measurement time preoperatively was 5.64±1.32 months (3.1 to 7.3 months). Postoperative UDVA was 0.07±0.10 (log MAR) and CDVA was -0.05±0.06 (log MAR). Postoperative SE was -0.17±0.18 D (Table 1).

**Table 1:** Study population characteristics.

Parameter	Value
Age (mean±SD, year)	66.16±10.37
Timespan (mean±SD, month)	5.64±1.32
Sex (female, %)	46.7%
Eye (right, %)	54.7%
Postoperative UDVA (mean±SD, log MAR)	0.07±0.10
Postoperative CDVA (mean±SD, log MAR)	-0.05±0.06
Postoperative SE (mean±SD, D)	-0.17±0.18

UDVA = uncorrected distance visual acuity; CDVA = corrected distance visual acuity; SE = spherical equivalent.

Comparisons of ocular parameters between the two time points were revealed (Table 2). The two time points showed no significant differences in AL, mean K and corneal astigmatism (P>0.05). However, the two time points showed statistical difference in ACD, IOL power, UDVA and CDVA during the average of 5.6 months before surgery (P<0.01). ACD decreased from 3.10±0.41 mm to 3.00±0.39 mm (P<0.05), and both UDVA and CDVA were significantly worse compared with the first time point (P<0.01).

**Table 2:** Ocular parameters at early period and 1 day preoperatively.

	AL (mm)	Mean K (D)	ACD (mm)	CA (D)	IOL power (D)	UDVA (logMAR)	CDVA (logMAR)
Early period	23.36±0.89	44.06±1.63	3.10±0.41	0.68±0.21	21.26±2.15	0.57±0.20	0.36±0.13
	22.10–24.45	41.16–47.17	2.35–3.83	0.24–0.87	15.5–25.5	0.24–0.92	0.05–0.5
Pre-1day	23.39±0.87	44.18±1.65	3.00±0.39	0.60±0.29	20.91±2.22	0.63±0.21	0.44±0.17
	22.16–24.38	41.34–47.04	2.37–3.73	0.22–0.91	15–25	0.25–1.0	0.05–0.7
P Value	0.31	0.57	**0.02**	0.27	**<0.01**	**<0.01**	**<0.01**

AL = axial length; K = keratometry; ACD = anterior chamber depth; CA = corneal astigmatism; IOL = intraocular lens; D = diopter; UDVA = uncorrected distance visual acuity; CDVA = corrected distance visual acuity; logMar = logarithm of the minimum angle of resolution; Comparisons between the two groups by a paired t test; P value < 0.05 was considered statistically significant.

Table 3 shows the differences between the MNE and MAE. The MNE at early period was -0.12±0.36 D and Pre-1day was -0.01±0.60 D. No statistically significant difference was detected in MNE (P>0.05). The MAE, 0.29±0.20 D and 0.50±0.34 D respectively, had a significant difference during the two time points (P<0.05).

**Table 3:** Postoperative refractive outcomes.

	MNE (D)	MAE (D)
Early period	-0.12±0.36	0.29±0.20
	-0.39–0.82	0.02–0.82
Pre-1 day	-0.01±0.60	0.50±0.34
	-0.95–1.08	0.03–1.08
P Value	0.12	**<0.01**

MNE = mean numerical error; MAE = mean absolute error; P value < 0.05 was considered statistically significant.

**Fig 1:**
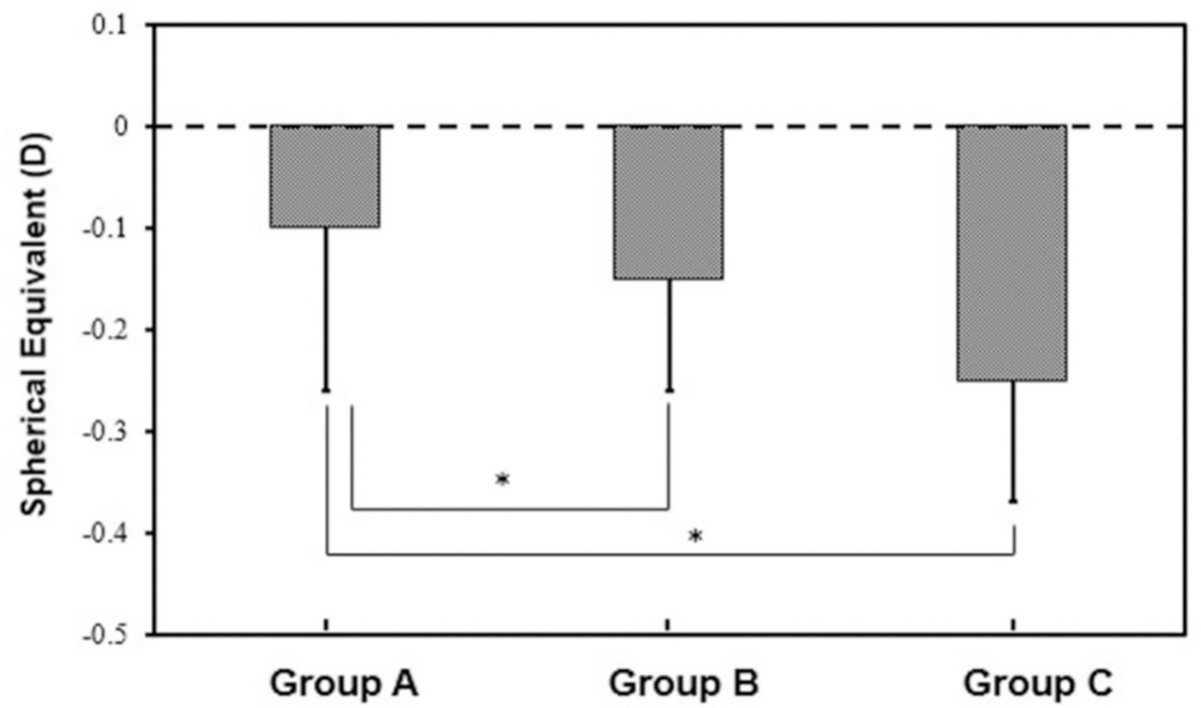
Postoperative spherical equivalent in the three groups divided by changes of the anterior chamber depth (ACD).

According to the change value of preoperative ACD, samples were divided into 3 groups: group A (ACD change value≤0.05 mm), group B (0.05 mm<ACD change value≤0.10 mm) and group C (ACD change value>0.10 mm). Postoperative SE varied among the 3 sub-groups based on different changes in ACD (Fig 1). Group A, B and C included 23, 28, 24 eyes, respectively, and changes of SE were -0.10±0.16 D, -0.15±0.11 D and -0.25±0.12 D. There were significant differences between Group A and C (P<0.05), Group B and C (P<0.05).

## Discussion

It is an essential procedure for ophthalmologist to predict the postoperative refractive outcome of cataract surgery. The Haigis formula, a fourth-generation formula, is a theoretical formula for calculating IOL power by AL, K and ACD [13]. Currently, the Haigis formula is utilized all over the world. In the Haigis formula, the estimation of ELP has not only the generality of large sample regression formulas, but the individualization of the formulas through individual measurement of ACD and AL. According to the Haigis formula, Haigis studied postoperative refraction of 108 eyes that underwent cataract surgery and found that postoperative refraction was predicted correctly within ± 1.0 D in 85.7% and within ± 2.0 D in 99% of all cases [14]. However, preoperative ACD became shallower with the degree of cataract progression, resulting in differences in the IOL power measured at two time points before surgery in clinical works. Differences in the accuracy of IOL power calculated by Haigis formula may be caused by ACD values.

In this study, ACD became shallower over time before surgery, which was consistent with the progression of cortical cataract. Chen found that in eyes with age-related cataract, lens thickness increased with an increase in cortical opacity, whereas the ACD and anterior chamber volume (ACV) values decreased [15]. It was reported that there was no statistically significant difference in AL, K and IOL power within the 6-month period using the IOL-Master [16]. However, the limitation of that study was that ages of subjects ranged from 28 to 87 years old, therefore lens difference of subjects was large. Ken proposed that age should be considered in the process of selecting IOL power, as ACD decreased with increase in age [17]. Similarly, the findings of this study showed that preoperative measurement time should be considered in calculation of IOL power. Nearly 45.3% patients showed a 0.25 D predicted error during the period, although this bias might be acceptable in clinic with a monofocal IOL.

The decreased ACD was the main factor causing the change of IOL power, resulting in refractive error of Haigis formula-calculated IOL power. The main source of error was the preoperative prediction of the postoperative position of the IOL, termed as ELP. Although ELP is related to the true IOL position, it is a fictitious position that helps guide the desired result [18]. On the basis of the Haigis formula [11, 13, 19], the IOL power will be smaller with the decrease of ACD, which was consistent with the findings of our study. It was reported that every 1.0 mm erroneous measurement of ACD can result in 1.5 D of refractive error [20]. In our study, the average ACD reduced by 0.1 mm and the MAE changed by 0.21 D. Among the results, the maximum change value of ACD was 0.6 mm, the related IOL power changed by 1.0 D, and the postoperative SE was -1.0 D supporting Olsen’s conclusion [20]. In order to reduce the error caused by device measurement, all measurements were taken three times by one experienced examiner and the data was averaged. Analysis of results of all the measurement data showed that only ACD changed in the 5 months period, therefore the error caused by the measurement can be excluded. Decrease in ACD may related to senile intumescent cataract, which is the most common cause among older individuals. In addition, the traditional view of the old people in China is that patients only accept surgery when the cataract seriously affects their vision, which may contribute to this reduction. Yi Chen et al. observed a reduction of ACD of about 0.1mm in patients whose cataracts were in the early to moderate stages [21]. However, he did not investigate the relationship between time and cataract development. A longitudinal study should be carried out to explore the relationship between ACD and aging [22]. The findings of our study showed that the IOL-Master examination in advance at early stages of age-related cataract can provide accurate biological parameters for cataract surgery, and the postoperative refractive state may result closer to emmetropia.

Although CDVA and UDVA changed significantly, the degree of cortical opacity may not affect ocular biometry parameters when LOCS III grade P ≤ 3.5. Previous studies found that the LOCS III opalescence score and lens density were positively correlated with CDVA [23, 24]. Tetsuo et al. reported that cataract progression or aging affected the refractive index of the lens, leading to the change in mean group refractive index, which ultimately resulted in inaccurate AL measurements [8]. Zhu et al. found that the degree of lens opacity correlated with poor preoperative biometry fixation stability, and that poor fixation contributed to AL errors, eventually causing postoperative refractive errors [25]. In this study no change in AL was observed, which may be because opacification of cataract was not severe enough to affect the measurements [23, 26]. This study was designed mainly to focus on early-to-moderate stage of age-related cataract patients and problems associated with cataract development that could have been overlooked before. Different types of cataracts might cause varying results. In addition, the severity of subcapsular cataract ranges from 3.5 to 5.0 leading to axial length measurements failure with the IOL-Master [27]. Therefore, patients with nuclear and posterior subcapsular cataracts were not included in our study. Future studies with different types of cataracts data should be carried out to further explore effects of nuclear and posterior subcapsular cataracts.

The MAE, 0.29±0.20 D at early period and 0.50±0.34 D at 1 day before surgery varied significantly at the two time points (P<0.05). Both MNE and MAE are the most used parameters to predict postoperative refractive error in cataract-related studies. Although the MAE is often used as a factor for the prediction accuracy of the IOL power, it does not show a direction of refractive outcomes (myopic or hyperopic). Therefore, MAE is usually used together with MNE to predict refractive errors [28]. In the present study, the difference of MNE for the two time points was not statistically significant. The small standard deviation of the MAE for the first measurement indicated that the accuracy of the prediction was better for the result measured at earlier period preoperatively. Although, the mean difference of IOL power (approximately 0.35 D) was very small, the findings of this study was still meaningful in the clinic, especially for those patients whose difference between two measurements were bigger than 0.50 D. Refractive accuracy is an important determinant for successful cataract surgery, therefore, advances in surgical technique and preoperative measurements result in increased accuracy [29]. We wish this exploration could give guidance to improve postoperative refraction outcomes in the future.

This study had a few limitations. First, patients with corneal astigmatism higher than 1.0 D were not included in this study. We excluded these patients because some patients choose to correct corneal astigmatism with implantation of Toric IOLs to achieve spectacle independence for distance vision after surgery. In addition, the incision location designed for the Toric lens implantation causes axial errors and introduces new variables, which would have affected the results [30]. Various designs of intraocular lenses should be explored in the future. Second, the study only included patients with cortical age-related cataract. Notably, people above 50 years old have a certain degree of nuclear opacity [31]. However, this phenomenon may not affect the results of our study. Chen et al. reported that ACD decreased with both nuclear and cortical cataracts, and the ACD changed significantly in eyes with cortical cataract compared with nuclear cataracts [32]. Further studies should compare different types of cataracts to verify and clarify this finding.

In conclusion, this study was conducted to explore the effect of cataract progression on accuracy of IOL power measurement. The correct preoperative time when the IOL power is calculated should be determined to optimize postoperative refractive outcomes. We found that ACD decreased with cataract progression and IOL power also changed before the surgery. The IOL power measured at earlier period preoperatively was more accurate and the postoperative refractive outcome tended to be emmetropia. Earlier measurement of IOL power at the initial stage of cataract may improve refractive results.

## Supporting information

S1 Checklist PLOS ONE clinical studies checklist. (DOCX)

S2 Checklist STROBE statement—checklist of items that should be included in reports of observational studies. (DOCX)
